# Genome wide analysis of meiotic recombination in yeast: For a few SNPs more

**DOI:** 10.1002/iub.1877

**Published:** 2018-06-22

**Authors:** Parijat Chakraborty, Ajith V. Pankajam, Abhishek Dutta, Koodali T. Nishant

**Affiliations:** ^1^ School of Biology Indian Institute of Science Education and Research Thiruvananthapuram Trivandrum India; ^2^ Centre for Computation Modelling and Simulation Indian Institute of Science Education and Research Thiruvananthapuram Trivandrum India

**Keywords:** meiosis, recombination, yeast, next generation sequencing, hybrid genome

## Abstract

Diploid organisms undergo meiosis to produce haploid germ cells. Crossover events during meiosis promote genetic diversity and facilitate accurate chromosome segregation. The baker's yeast *Saccharomyces cerevisiae* is extensively used as a model for analysis of meiotic recombination. Conventional methods for measuring recombination events in *S. cerevisiae* have been limited by the number and density of genetic markers. Next generation sequencing (NGS)‐based analysis of hybrid yeast genomes bearing thousands of heterozygous single nucleotide polymorphism (SNP) markers has revolutionized analysis of meiotic recombination. By facilitating analysis of marker segregation in the whole genome with unprecedented resolution, this method has resulted in the generation of high‐resolution recombination maps in wild‐type and meiotic mutants. These studies have provided novel insights into the mechanism of meiotic recombination. In this review, we discuss the methodology, challenges, insights and future prospects of using NGS‐based methods for whole genome analysis of meiotic recombination. The objective is to facilitate the use of these high through‐put sequencing methods for the analysis of meiotic recombination given their power to provide significant new insights into the process. © 2018 The Authors. IUBMB Life published by Wiley Periodicals, Inc. on behalf of International Union of Biochemistry and Molecular Biology, 70(8):743–752, 2018

**Abbreviations**
BAM: binary alignment map
ChIP: chromatin immunoprecipitation
cM: centimorgan
DSB: double strand break
DNA: deoxyribonucleic acid
IGV: integrated genomics viewer
NGS: next generation sequencing
QC: quality control
SAM: sequence alignment map
SDSA: synthesis dependent strand annealing
SSN: structure selective nuclease
SNP: single nucleotide polymorphism
VCF: variant calling format

## INTRODUCTION

All sexually reproducing organisms undergo two rounds of division (Meiosis I and Meiosis II) to produce haploid gametes from diploid progenitor cells. Homologous recombination events during Meiosis I, such as crossovers, non‐crossovers and gene conversions generate genetic diversity. In addition, crossovers facilitate disjunction of homologous chromosomes during Meiosis I by promoting physical linkages between the homolog pairs that oppose the spindle generated forces pulling the homologs apart. The opposing forces provide the tension necessary for the correct alignment and disjunction of the homologous chromosomes 1. The number and spatial distribution of crossovers are tightly regulated to ensure at least one crossover per homolog pair. Segregation errors in meiosis results in aneuploidy, which is a major cause of genetic birth defects in humans 2.

**Figure 1:**
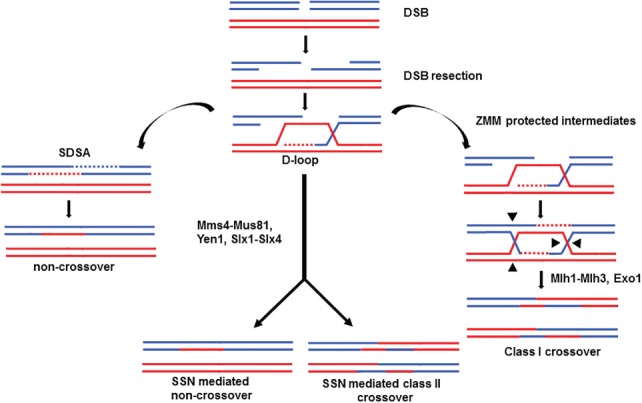
Recombination outcomes of DSB repair pathways during meiosis. DSB resection and strand invasion leads to D‐loop formation. Dissolution of the D‐loop results in non‐crossovers by SDSA mechanism. Protection of D‐loops from disassembly by the ZMM proteins allow double Holliday junction formation that is acted on by Mlh1‐Mlh3 and Exo1 to form the class I crossovers. Alternatively the joint molecules, may also be processed by the structure selective nucleases Mms4‐Mus81, Slx1‐Slx4 and Yen1 to form the class II crossovers and non‐crossovers.

The baker's yeast *Saccharomyces cerevisiae,* has been used extensively as a model organism to study meiosis. *S. cerevisiae* can undergo meiotic divisions rapidly (∼12 h for SK1 strain). Further, the small genome size of *S. cerevisiae* (12 Mb) and the ease of genetic modification facilitate a wide array of experimental analysis. In *S. cerevisiae*, crossovers are initiated by the formation of 140–170 double strand breaks (DSBs) by a conserved type II topoisomerase Spo11 along with accessory factors 3, 4. Meiotic DSB repair, preferentially using the homolog as a template results in either a crossover or non‐crossover outcome (Fig. 1). During DSB repair, the invading strand may get displaced from the homolog and ligate with the opposite end of the break leading to the formation of non‐crossovers by synthesis dependent strand annealing (SDSA) pathway 5. If the strand invasion is stabilized by the ZMM proteins (Zip1, Zip2, Zip3, Zip4, Mer3, Msh4, Msh5 and Spo16), it may be extended further by repair synthesis using the homolog and capture the second end of the DSB and form double Holliday junction 6. Biased resolution of these double Holliday junctions facilitated by the ZMM, STR (Sgs1, Top3, Rmi1), Exo1 and the Mlh1‐Mlh3 endonuclease leads to crossovers 7, 8, 9, 10, 11, 12, 13, 14. These class I crossovers show interference—a phenomena where the occurrence of a crossover event in a genetic interval makes it less likely for crossovers to occur in adjacent intervals. Another set of crossovers (Class II) are generated from the Holliday junction intermediates by Mms4‐Mus81, Yen1, and Slx1‐Slx4, the structure selective nucleases (SSNs). Resolution by the SSNs lack crossover bias, and both crossovers and non‐crossovers are produced. These class II crossovers do not show interference 15, 16.

Classical genetics, cytological methods, and physical analysis, have been conventionally used to characterize meiotic recombination in *S. cerevisiae*. In this review, we describe the advantages of next generation sequencing (NGS)‐based methods for genome wide analysis of meiotic recombination compared to these conventional methods of recombination mapping. We also discuss issues related to the experimental and bioinformatics aspects of genome wide recombination analysis to make this comparatively new area more familiar to the researchers.

## CONVENTIONAL METHODS FOR ANALYSIS OF MEIOTIC RECOMBINATION IN *S. cerevisiae*

Classical genetic methods involve the use of auxotrophic or drug markers whose segregation can be visually monitored in meiotic spores to measure crossover frequency and gene conversions. The information from segregation of markers in meiotic spore progeny gives an estimate about the recombination frequency between those markers. This method is still popular as it is cost‐effective and provides a basic idea of the recombination frequency before initiating a more elaborate and expensive NGS‐based approach. The ease of genetic manipulation in *S. cerevisiae* means the markers can be inserted at the desired location in the genome to estimate the recombination frequency of the locus. These estimates are often extrapolated on a whole genome scale. For example in the *S. cerevisiae* SK1 strain, a 395 kb region in chromosome XV has been modified with six auxotrophic markers that corresponds to a genetic distance of 100.9 cM in wild‐type meiosis 17. To generate data from more loci, additional genetic markers were integrated into representative small, medium and large chromosomes (Chr III, VIII and VII, respectively) 15. The crossover defects in many meiotic mutants have been analyzed using these strains 12, 17, 18, 19, 20. The major drawback of the recombination maps generated from this method is that it requires the analysis of a large population of tetrads and provides locus specific data. Since recombination rates are not constant across the *S. cerevisiae* genome, locus specific measures may not accurately represent the genome‐wide recombination frequency. Another drawback is the dependence on spore viability making it difficult to analyze meiotic mutants with severe defects in viability or meiotic progression. Also only a limited number of auxotrophic or drug markers can be introduced.

Unlike classical genetic analysis, cytological methods involve immunostaining of crossover specific proteins like Zip3, Msh4/Msh5 on meiotic chromosome spreads. This method provides information on recombination frequency on a genome wide scale and is independent of the viability of the spores 13, 18. The number of foci correlates with an increase or decrease in the recombination events. The drawback of this method is that one has to rely on the proportional change of the foci number, which does not give a count of the actual number of recombination events and it also provides low resolution data. Another cytological method relies on the use of fluorescent tetrads to detect recombination events 21. This method bypasses the necessity of viable tetrads. The selected homolog pairs are marked in the appropriate loci with fluorescent markers and direct visualization of the tetrads under the microscope reveals the marker segregation pattern. This method can analyze crossovers, non‐crossovers, chromosome non‐disjunction without dissecting numerous tetrads 21. In spite of its advantages, the method provides locus specific data.

Besides genetic and cytological methods, single locus physical assays have been developed to quantify the recombination products in *S. cerevisiae*. A well‐characterized DSB hotspot (*e.g. HIS4‐LEU2* on Chromosome III) is modified with restriction enzyme sites to distinguish the homologous chromosomes 11, 12. The DNA from synchronized meiotic cultures are digested with the appropriate restriction enzymes. The products are analyzed by two‐dimensional gel electrophoresis and probed to identify DSBs, joint molecules—both interhomolog and intersister, crossover and non‐crossover products 11, 22. This method circumvents the issue of spore viability and has been used to study meiotic mutants with severe viability defects 10, 14, 15, 16, 23, 24, 25. But this method also has the limitation of reporting data of only one locus.

In summary, classical genetic analysis, and physical assays provide recombination information from a specific locus with good resolution, whereas cytology can provide genome wide recombination data but at low resolution. With NGS‐based methods, the trade off between resolution and genome coverage becomes irrelevant. But like any genetic method, recombination analysis by NGS requires the four spores to be viable, making it difficult to analyze mutants with poor spore viability or defects in meiotic progression. With advances in DNA sequencing techniques, the complete sequence of many *S. cerevisiae* strains are accessible in the *Saccharomyces* Genome Database (https://www.yeastgenome.org/). The presence of a wide variety of *S. cerevisiae* strains with sequenced genomes provides a multitude of strain combinations to generate hybrids. Sufficient density of single nucleotide polymorphisms in the hybrid strains allows us to map recombination events genome wide at high‐resolution using NGS analysis as described below.

## NGS‐BASED ANALYSIS OF HYBRID *S. cerevisiae* STRAINS TO MAP MEIOTIC RECOMBINATION GENOME WIDE

**Figure 2:**
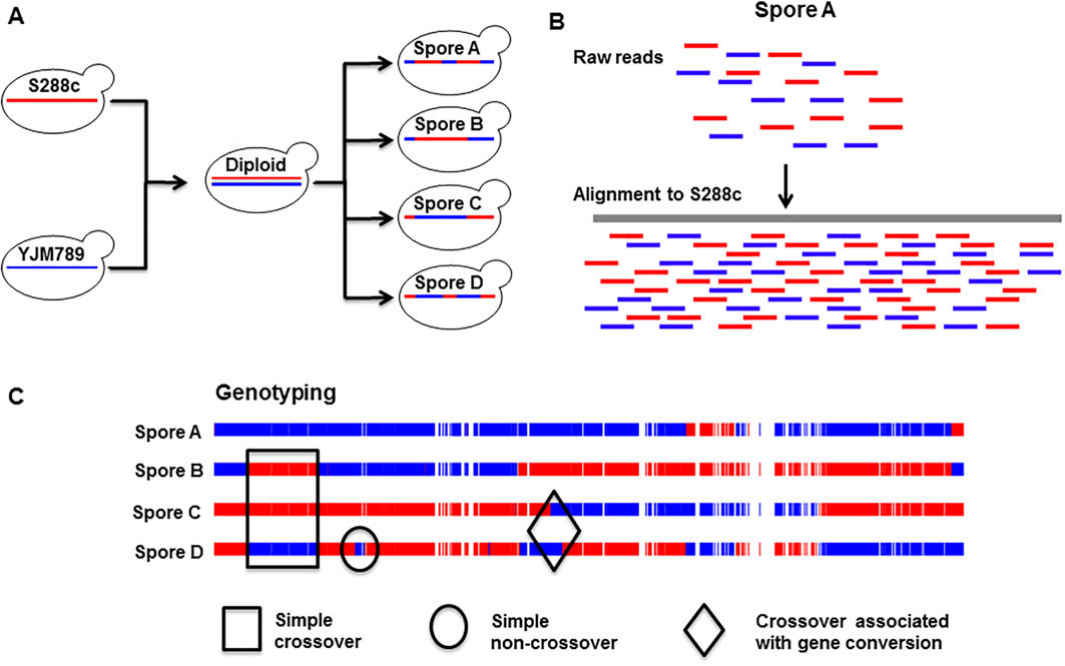
Recombination mapping using NGS analysis of hybrid yeast strains. (A) Representative cross involving the S. cerevisiae S288c and YJM789 strains to generate hybrid diploid. The diploid is sporulated and the spores are sequenced using NGS methods. (B) Alignment of whole genome sequence data from spores to a reference genome for calling variants (SNPs). (C) Recombination outcomes that may be detected from SNP segregation data in the four spores from a single tetrad. Rectangular box shows simple crossovers that can be identified by the reciprocal exchange of flanking markers in 2:2 segregation pattern. Diamond box shows crossovers accompanied by gene conversions with the segregation of markers in 1:3 or 3:1 ratio around the exchange sites. Circle shows simple non‐crossovers which can be detected by the presence of 1:3 or 3:1 segregation tracts without any exchange of flanking markers. In addition minority recombination outcomes caused by events like multiple chromatid invasions or exchanges involving more than two chromatids can also be detected by marker segregation patterns containing signatures unique to the mechanism.

Though high‐resolution genome wide recombination analysis is now routinely performed using NGS, it started with microarray based methods by Mancera et al. 26 using *S. cerevisiae* S288c/YJM789 hybrid (Fig. 2). The YJM789 strain is a clinical isolate and 0.6% diverged from the standard laboratory S288c strain 27. DNA isolated from four viable spore tetrads of the hybrid were fragmented, fluorescently labeled and hybridized against the microarray that contains probes for both the parental SNPs at 4 bp resolution. Around 52,000 SNPs were called which were uniformly distributed throughout the genome with an average inter‐marker distance of 78 bp 26. A similar study by Chen et al. called 8,000 markers between the two parental strains (S288c, YJM789) with an average distance of 1.5 kb between two consecutive markers 28. Both the studies reported almost similar number of crossovers (90.5 and 95), but non‐crossovers 19 were fewer in Chen et al. (28) compared to the non‐crossovers (66) from Mancera et al. (26). This is because lower SNP density affects the detection of non‐crossovers but not crossovers. Microarray‐based analysis of genome wide recombination data has largely given way to NGS‐based methods due to falling sequencing costs as well as some of the limitations of microarrays (Fig. 2). These include (i) requirement of prior knowledge of the genome sequence of the organism to design oligo probes for hybridization. (ii) Single nucleotide resolution provided by NGS methods compared to microarray based methods where the resolution is dependent on the probe density. (iii) Potential for errors due to false hybridization in microarray based methods. Below we discuss some of the issues involved in the design of experimental and bioinformatics methods for genome wide recombination mapping in hybrid yeast using NGS.

### Choice of the Appropriate Yeast Hybrid and Marker Density

The small size of the *S. cerevisiae* genome (12 Mb) and the availability of the genome sequences of various *S. cerevisiae* strains have made genome wide recombination analysis easier and cheaper compared to other organisms having complex, large genomes. The presence of a sufficient number of uniformly distributed SNP markers is a requisite for high‐resolution recombination mapping. But a very high density of SNP markers may not be advantageous as the SNPs are treated as mismatches during DSB repair using the homolog. High sequence divergence may cause the mismatch repair response to reject strand invasion into the homolog. This may result in recombination outcomes that favor sister chromatid repair as well as complex recombination outcomes that are difficult to detect 29, 30. For example, Martini et al. have shown that in a cross between *S. cerevisiae* SK1 and S288c strains (0.7% divergence), 73 crossovers are made 30. Deletion of the mismatch repair gene, *MSH2* in both the parents increased the crossovers to 92 (30). Hybrids with higher SNP density also show a significant drop in viability (Table 1), suggesting a negative correlation of spore viability with heterozygosity 30, 32, 33, 34. So, choosing the appropriate hybrid is important. Hybrid choice should be determined by the evolutionary origin of the strains. The *S. cerevisiae* SK1 strain has evolved separately from all other strains used in the lab. As a result higher sequence divergence, reduced viability and recombination is observed in crosses involving SK1 with other *S. cerevisiae* strains (Table 1). On the other hand, *S. cerevisiae* strains like S288c, RM11, YJM789 etc., are of similar origin 35, 36. Crosses involving these strains like S288c/YJM789 or RM11/S288c show better spore viability and have similar crossover frequency as the well‐characterized isogenic SK1 strain (Table 1). An important advantage of using the S288c/YJM789 hybrid is its non‐mutagenic nature. The mitotic base mutation rate of this hybrid (1.82 × 10^−10^ per base per division) is almost similar to that of the isogenic S288c and SK1 strains 34.

**Table 1:** S. cerevisiae artificial hybrids

Hybrids	SNPs	Crossovers	Spore viability (%)
S288c/YJM789 (26, 31)	52,000	90.5	84
RM11‐1a/YJM789 (32)	30,000	NA	90
S288c/RM11‐1a (33)	46,000	91	85
S288c/SK1 (30)	62,000	73	70
SK1/YJM789 (34)	65,000	NA	77
SK1/RM11‐1a (34)	69,000	NA	76

The approximate number of SNPs, average crossovers and spore viability of different hybrids is shown.

### Sequencing and Phasing of SNPs

In principle, any NGS method can be used to genotype SNPs in the meiotic spores for recombination mapping. Sequencing techniques with shorter reads like Illumina (https://www.illumina.com) increase the chance of misalignment in the repeat sequences. So, the telomeric regions, Ty element containing sequences etc are excluded from the SNP analysis when using such technology. Sequencing technologies with longer reads like PacBio (https://www.pacb.com) or nanopore (https://nanoporetech.com) are more useful if SNPs from repeat regions need to be analyzed for recombination data. New mutations that are heterozygous can arise while the spore cultures are grown for several generations to isolate DNA. Although genotyped, these are usually ignored and not used for downstream recombination analysis. It is therefore useful to sequence the parent strains available in the lab so that new mutations in spore DNA can be identified and eliminated from the analysis or used if they are present in the parent as well.

Whole genome recombination mapping requires the knowledge of the phases of the SNP markers, that is, the concerned marker belongs to which parent. Without phasing, the segregation analysis of the SNP markers in the spores is not possible. For standard artificial hybrids like S288c/YJM789, SK1/S288c or RM11/S288c, the reference genomes of the parent strains are available. Therefore the phases of the SNP markers are known and any sequencing method, even those with short reads (*e.g*., Illumina) is fine. But in nature, many yeast hybrids exist and to understand meiotic recombination in a natural hybrid can be of great value. But the problem of phasing of the SNP markers arises. To overcome this problem, techniques (PacBio, nanopore), which give very long reads, are useful since SNPs frequently occurring together in a single read are likely to be in phase. With long reads (in kbs), the alignment will be easier, and all the SNP markers can be phased 37, 38.

### Bioinformatic Analysis of SNP Segregation and Recombination

**Figure 3:**
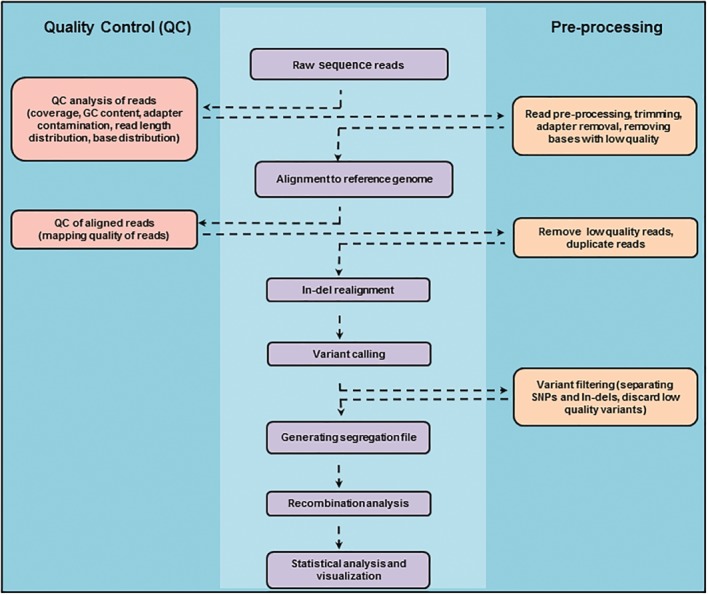
Flowchart of the bioinformatics analysis for inferring recombination events from whole genome sequence data of meiotic spores.

The computational analysis of SNP segregation and recombination from Illumina short sequence reads involves multiple steps (Fig. 3).

#### *Quality Control (QC) Analysis, Preprocessing and Coverage*

Raw sequence reads that are generated from the Illumina platform are in fastq file format which includes the quality information of the bases. It is critical to thoroughly check the base qualities and read statistics (number of reads, overall coverage, base distribution, GC content, over represented reads, adapters contamination, read duplication, etc.) for the samples that were sequenced. Low quality bases at the ends of the reads are trimmed and sequencing adapters and artifacts are removed. The minimum coverage required varies for different types of sequencing projects. For mapping recombination events, we analyze the change in a set of consecutive markers, since a crossover or conversion tract involves multiple markers. Therefore the probability of all the markers within a tract being genotyped incorrectly due to sequencing error is low. So each SNP coverage can be lower (∼10×) compared to the coverage required for mutational analysis. Paired end reads are preferred over single end reads for better accuracy in mapping the reads to a reference genome and genotyping. Some of the widely used softwares to check the statistics and the quality of bases in the reads are listed in Table 2.

**Table 2:** Widely used open source softwares for bioinformatics analysis of NGS data

Module and specific tool	Website link
Quality control analysis	
FastQC	https://www.bioinformatics.babraham.ac.uk/projects/fastqc/
NGS QC Toolkit	http://www.nipgr.res.in/ngsqctoolkit.html
Pre‐processing of reads	
Trimmomatic	http://www.usadellab.org/cms/?page=trimmomatic
FASTX‐Toolkit	http://hannonlab.cshl.edu/fastx_toolkit/
Alignment to reference genome	
Bowtie2	http://bowtie-bio.sourceforge.net/ bowtie2/ index.shtml
BWA MEM	http://bio-bwa.sourceforge.net/
Stampy	http://www.well.ox.ac.uk/project-stampy
Removal of duplicate reads	
Picard (Markduplicates)	http://broadinstitute.github.io/picard/
Samtools (rmdup)	http://samtools.sourceforge.net/
Indel realignment	
GATK (IndelRealignment)	https://software.broadinstitute.org/gatk/
Genotyping	
GATK (HaplotypeCaller/UnifiedCaller)	https://software.broadinstitute.org/gatk/
Samtools (mpileup)	http://samtools.sourceforge.net/
Recombination analysis	
ReCombine	https://sourceforge.net/projects/recombine/
Statistical analysis and visualization	
R	https://www.r-project.org/
IGV	http://software.broadinstitute.org/software/igv/

#### *Read Alignment*

After QC analysis and preprocessing, the reads are aligned to the reference genome. The result of the aligned reads are in SAM (sequence alignment map) format which may be compressed to yield BAM (binary alignment map) files. BAM files contain information regarding the read location in the reference genome and also some additional information (eg mapping quality, uniquely represented reads, duplicated reads, etc). Duplicate reads detected from the alignment should be removed as they may skew the allele frequency and lead to false genotyping. Indel realignment facilitates removal of alignment artifacts which reduce the accuracy of genotyping. If the percentage of mapped reads from the alignment is less, we may be able to call fewer SNPs. For example, if a sample contains around 8,000 SNPs (*i.e*. 1 SNP per 1.5 kb) then that sample can detect crossovers but not some gene conversions that often have a median length of 1.5 kb 28.

#### *Genotyping and Generation of Segregation Files*

Multi‐sample genotyping or variant calling is preferred. This involves calling variants across multiple samples (spores) at a given location, if, any one of the samples contains a variant in the same location. Multi‐sample variant calling reduces the computational load and is easier for a small genome like that of *S. cerevisiae*. This step returns a file containing variants called VCF (variant calling format). Custom scripts (any programming language) are used to generate segregation files by clubbing VCF files of genotyped spores as tetrads.

#### *Recombination Analysis*

For recombination analysis, the software should correctly identify and classify the recombination events from the segregation file. Mancera et al. used the ssGenotyping program to identify the crossover and non‐crossover events from the microarray data 26. Anderson et al. developed ReCombine package (written in python) that can analyze recombination events from microarray or NGS data from yeast tetrads 39. This program takes processed fastq files, genotypes and generates segregation files. The CrossOver program in the ReCombine package can use the segregation file to detect crossover, non‐crossover and gene conversion events and classify them into categories 39. By default, ReCombine merges adjacent crossovers or a crossover, non‐crossover event if they are separated by less than 5 kb as they are likely to have initiated from the same DSB. This parameter can be adjusted. The drawback of ReCombine is that it may call discontinuous non‐crossover tracts from the same initiating DSB as separate events thereby skewing the non‐crossover numbers 29. To avoid this problem CrossOver may be run with 0 kb cut off to identify all the crossover and non‐crossover tract changes as individual events 29. This is then used as an input for “groupEvents” program, which groups the crossover and non‐crossover events, lying within 5 kb range, together as a single event 29. These modifications can be used to analyze the complexities of the non‐crossover conversion tracts. So adjacent discontinuous non‐crossover tracts caused by multiple strand invasions from the same initiating DSB which would have been called as two separate events in Crossover program will be now merged as a single non‐crossover event using the groupEvents script 29. The “groupEvents” program enabled Oke et al. 2014 (29) to accurately characterize conversion tracts in meiotic mutants and detect complex recombination outcomes in yeast and the causal mechanisms 29. Although ReCombine makes recombination mapping in yeast easy, it can only generate segregation files with crosses involving S288c. For other yeast hybrids, the program has to be modified to align and call the SNPs from a different reference genome. Alternatively, the segregation file can be separately generated and used as input for the CrossOver module in ReCombine.

#### *Non‐detectable Recombination Events*

Although NGS analysis of spores from hybrid strains can facilitate high‐resolution genome wide mapping of recombination events, it is important to note that not all recombination events can be detected. The DSBs are repaired using either the homolog or the sister chromatid as a template. In wild‐type meiosis, interhomolog recombination is preferred over intersister events 40, 41. In many mutants like *pch2*, *mek1*, etc. the bias is affected, and the intersister events increase 42, 43, 44. NGS‐based recombination analysis requires heterozygous SNPs, so only the interhomolog events are detected. The lack of markers on the sister chromatids makes it difficult to quantify inter‐sister recombination events, especially in mutants where these may be elevated. As a consequence overall recombination events are under‐estimated and may not correlate with DSB frequency. The Strand‐seq technique was recently used to map inter‐sister exchange events genome‐wide during mitosis 45, 46 in *S. cerevisiae*. Such methods could be further developed to possibly detect inter‐sister repair events during meiosis. Yeast hybrids often have high marker densities (*e.g*. 1 SNP every 78 bp in S288c/YJM789 hybrid), which facilitates detection of all crossover events, since they affect flanking markers. But it is possible that non‐crossover events that occur in between adjacent markers are not detected 26.

#### *Statistical Analysis, Visualization and Data Management*

All statistical analysis and visualization may be performed using any statistical analysis package (*e.g*. R, Matlab, etc.). The data can be also visualized with many open source softwares like UCSC genome browser, Integrated Genomics Viewer (IGV) etc. (Table 2). These provide the user an interactive analysis tool at a single base pair scale 47, 48, 49. Data management and analysis are challenging since millions of short reads amounting to terabytes of data are generated from whole genome sequencing of spores. Error free SNP calling requires the genome to be sequenced at high coverage (10× or greater). For statistical significance in the data, sufficient numbers of tetrads are sequenced to obtain a reliable count of crossover/non‐crossover numbers. These generate a huge amount of sequence data even for a single experiment. Storing the raw data requires sufficient space (∼2–3 GB per haploid yeast genome at 30× coverage).

### Correlating Data from Hybrids with Isogenic Strains

High‐resolution recombination mapping in yeast is built on the use of hybrids. Apart from issues like sequence divergence and incompatibilities, the asynchrony in the meiotic kinetics of hybrids poses problems. Asynchrony does not affect recombination mapping in hybrid strains, as the tetrads are specifically selected. But other techniques like immunofluorescence analysis or ChIP sequencing requires the sporulating culture to be highly synchronous as the analysis at a particular time point should reflect the state of the majority of the cell population. So, these techniques cannot be used in hybrids to compare with the genetic recombination data obtained from NGS analysis. Instead, isogenic strains like SK1, which shows high synchrony in meiosis, are used for cytological and ChIP‐sequencing studies and the data are correlated with the recombination analysis in hybrid strains. A disadvantage of this approach is the assumption that the isogenic and the hybrid strains have similar properties of meiotic recombination, which may not be the case 50. In addition, meiotic mutants may show differences in sporulation efficiency and spore viability in the hybrid compared to isogenic strains. For example, *mms4*Δ sporulates in isogenic SK1 and shows 46%–51% viability 15, 20, but it fails to sporulate in S288c/YJM789 hybrid.

## NEW INSIGHTS FROM GENOME WIDE ANALYSIS OF MEIOTIC RECOMBINATION

Genome‐wide fine‐scale mapping of recombination using hybrid *S. cerevisiae* genomes have provided new insights into the mechanisms of genetic recombination in eukaryotes. The initial study by Mancera et al. (26) using the S288c/YJM789 hybrid, provided the first genome wide recombination map in yeast with many new mechanistic insights, such as the presence of non‐crossover hotspots in the genome, interference between crossovers and non‐crossovers etc. A similar study by Qi et al. using a different hybrid (S288c/RM11) described crossover, non‐crossover and associated gene conversion tracts at single base resolution in wild type *S. cerevisiae* 33. Further studies by Mancera et al. 51 provided information on the genome wide distribution and prevalence of post meiotic segregation events. Similarly, genome wide analysis of various meiotic mutants have revealed novel functions of meiotic genes. For example, a role for Zip1 in suppressing crossing over at the centromere 28; the effect of diminishing recombination initiation in *spo11* hypomorphs on DSB repair outcome 52; a role for Zip3 in biased resolution of Holliday junctions into crossovers and a role for Mms4 in suppressing multiple strand invasions during DSB repair 29. Genome wide mapping of recombination in *tel1* mutants showed loss of crossover interference and a role for Tel1 in regulating DSB distribution along the chromosome 53. Genome wide recombination mapping of *msh2*Δ elucidated barriers mismatch repair poses to recombination as well as new models for recombination 30. Similarly genome wide recombination mapping in *mlh2*Δ mutants showed a role for Mlh1‐Mlh2 in regulating the extent of gene conversion tracts 54. Genome wide recombination mapping in *msh4* hypomorphs that make fewer crossovers but have normal viability, supported a role for crossover distribution mechanisms in ensuring the obligate crossover 55. Genome wide recombination mapping in *pch2* mutants revealed increased crossovers and non‐crossovers as well as loss of chromosome size dependent DSB formation 31. Another study in a series of *mlh3* point mutants showed a genome wide increase in non‐crossovers that supports a structural role for this complex in deciding the fate of meiotic recombination intermediates 56. Furthermore, recent studies in alternate yeast models such as *Lachancea kluyveri* hybrid have shed light on the significant inter‐specific variation in meiotic recombination frequency 57. Whole genome analysis of *L. kluyveri* meiotic spores revealed lower crossover frequencies compared to *S. cerevisiae*, a high proportion of non‐exchange chromosomes as well as a high frequency of 4:0 conversion tracts in the hybrid indicative of the role of abortive meiosis in genome evolution 57, 58. These above mentioned studies are a few examples of the new insights into meiotic recombination mechanisms using genome wide recombination mapping methods.

## FUTURE PROSPECTS

Apart from yeast, genome wide analysis of meiotic recombination is also being used in other species like humans, *Arabidopsis*, maize, etc. 59, 60, 61, 62. There are a few areas where further developments may overcome some of the limitations in using NGS analysis for mapping recombination events.

In artificial *S. cerevisiae* hybrids like S288c/YJM789 many meiotic mutants show poor viability. Use of other uncharacterized hybrids or natural hybrids may address the poor viability issue of some meiotic mutants in a hybrid context. Another approach is to develop methods for sequencing the spores directly without germinating them. With the advent of single cell sequencing technology, the spores isolated from the tetrads can be lysed and the DNA amplified from each spore can be sequenced to map recombination events. This method also has the additional advantage that it eliminates selection bias towards viable spores.

Making use of the NGS data requires multiple programs, most of which are command line based. Expertise in computational biology is necessary for the data analysis. As many biologists lack exposure in the computational field, it becomes difficult for them to analyze the whole genome data. To make the process more user‐friendly, a graphical interface could be developed instead of command lines, where the user could feed the segregation files as input and obtain the crossover, non‐crossover and gene conversion data. Such an advancement will make the data analysis considerably easy. Simplification of data analysis through user friendly and easy to use input output formats while maintaining accuracy need to be developed to promote wider acceptance of NGS in recombination analysis. These developments and newer NGS technologies will make whole genome recombination analysis more popular and accessible to the scientific community.

## References

[1] Petronczki, M. , Siomos, M. F. and Nasmyth, K. (2003) Un menage a quatre: the molecular biology of chromosome segregation in meiosis. Cell, 112, 423–440. 12600308 10.1016/s0092-8674(03)00083-7

[2] Hassold, T. and Hunt, P. (2001) To err (meiotically) is human: the genesis of human aneuploidy. Nat Rev Genet, 2, 280–291. 11283700 10.1038/35066065

[3] Keeney, S. , Giroux, C. N. and Kleckner, N. (1997) Meiosis‐specific DNA double‐strand breaks are catalyzed by Spo11, a member of a widely conserved protein family. Cell, 88, 375–384. 9039264 10.1016/s0092-8674(00)81876-0

[4] Lam, I. and Keeney, S. (2014) Mechanism and regulation of meiotic recombination initiation. Cold Spring Harb Perspect Biol, 7, a016634. 25324213 10.1101/cshperspect.a016634 PMC4292169

[5] McMahill, M.S. , Sham, C.W. and Bishop, D.K. (2007) Synthesis‐dependent strand annealing in meiosis. PLoS Biol, 5, e299. 17988174 10.1371/journal.pbio.0050299 PMC2062477

[6] Lynn, A. , Soucek, R. and Borner, G.V. (2007) ZMM proteins during meiosis: crossover artists at work. Chromosome Res, 15, 591–605. 17674148 10.1007/s10577-007-1150-1

[7] Manhart, C.M. , Ni, X. , White, M.A. , Ortega, J. , Surtees, J.A. and Alani, E. (2017) The mismatch repair and meiotic recombination endonuclease Mlh1‐Mlh3 is activated by polymer formation and can cleave DNA substrates in trans. PLoS Biol, 15, e2001164. 28453523 10.1371/journal.pbio.2001164 PMC5409509

[8] Allers, T. and Lichten, M. (2001) Differential timing and control of noncrossover and crossover recombination during meiosis. Cell, 106, 47–57. 11461701 10.1016/s0092-8674(01)00416-0

[9] Borner, G.V. , Kleckner, N. and Hunter, N. (2004) Crossover/noncrossover differentiation, synaptonemal complex formation, and regulatory surveillance at the leptotene/zygotene transition of meiosis. Cell, 117, 29–45. 15066280 10.1016/s0092-8674(04)00292-2

[10] De Muyt, A. , Jessop, L. , Kolar, E. , Sourirajan, A. , Chen, J. , Dayani, Y. and Lichten, M. (2012) BLM helicase ortholog Sgs1 is a central regulator of meiotic recombination intermediate metabolism. Mol Cell, 46, 43–53. 22500736 10.1016/j.molcel.2012.02.020 PMC3328772

[11] Hunter, N. and Kleckner, N. (2001) The single‐end invasion: an asymmetric intermediate at the double‐strand break to double‐holliday junction transition of meiotic recombination. Cell, 106, 59–70. 11461702 10.1016/s0092-8674(01)00430-5

[12] Nishant, K.T. , Plys, A.J. and Alani, E. (2008) A mutation in the putative MLH3 endonuclease domain confers a defect in both mismatch repair and meiosis in Saccharomyces cerevisiae. Genetics, 179, 747–755. 18505871 10.1534/genetics.108.086645 PMC2429871

[13] Shinohara, M. , Oh, S.D. , Hunter, N. and Shinohara, A. (2008) Crossover assurance and crossover interference are distinctly regulated by the ZMM proteins during yeast meiosis. Nat Genet, 40, 299–309. 18297071 10.1038/ng.83

[14] Zakharyevich, K. , Tang, S. , Ma, Y. and Hunter, N. (2012) Delineation of joint molecule resolution pathways in meiosis identifies a crossover‐specific resolvase. Cell, 149, 334–347. 22500800 10.1016/j.cell.2012.03.023 PMC3377385

[15] de los Santos, T. , Hunter, N. , Lee, C. , Larkin, B. , Loidl, J. and Hollingsworth, N.M. (2003) The Mus81/Mms4 endonuclease acts independently of double‐Holliday junction resolution to promote a distinct subset of crossovers during meiosis in budding yeast. Genetics, 164, 81–94. 12750322 10.1093/genetics/164.1.81 PMC1462551

[16] Oh, S.D. , Lao, J.P. , Taylor, A.F. , Smith, G.R. and Hunter, N. (2008) RecQ helicase, Sgs1, and XPF family endonuclease, Mus81‐Mms4, resolve aberrant joint molecules during meiotic recombination. *Mol* Cell, 31, 324–336. 10.1016/j.molcel.2008.07.006 PMC2587322 18691965

[17] Argueso, J.L. , Wanat, J. , Gemici, Z. and Alani, E. (2004) Competing crossover pathways act during meiosis in Saccharomyces cerevisiae. Genetics, 168, 1805–1816. 15611158 10.1534/genetics.104.032912 PMC1448724

[18] Nishant, K.T. , Chen, C. , Shinohara, M. , Shinohara, A. and Alani, E. (2010) Genetic analysis of baker's yeast Msh4‐Msh5 reveals a threshold crossover level for meiotic viability. *PLoS Genet*, 6. 10.1371/journal.pgen.1001083 PMC2928781 20865162

[19] Zanders, S. and Alani, E. (2009) The pch2Delta mutation in baker's yeast alters meiotic crossover levels and confers a defect in crossover interference. PLoS Genet, 5, e1000571. 19629178 10.1371/journal.pgen.1000571 PMC2709914

[20] Sonntag Brown, M. , Lim, E. , Chen, C. , Nishant, K.T. and Alani, E. (2013) Genetic analysis of mlh3 mutations reveals interactions between crossover promoting factors during meiosis in baker's yeast. G3 (Bethesda), 3, 9–22. 23316435 10.1534/g3.112.004622 PMC3538346

[21] Thacker, D. , Lam, I. , Knop, M. and Keeney, S. (2011) Exploiting spore‐autonomous fluorescent protein expression to quantify meiotic chromosome behaviors in Saccharomyces cerevisiae. Genetics, 189, 423–439. 21840861 10.1534/genetics.111.131326 PMC3189805

[22] Schwacha, A. and Kleckner, N. (1995) Identification of double Holliday junctions as intermediates in meiotic recombination. Cell, 83, 783–791. 8521495 10.1016/0092-8674(95)90191-4

[23] Jessop, L. and Lichten, M. (2008) Mus81/Mms4 endonuclease and Sgs1 helicase collaborate to ensure proper recombination intermediate metabolism during meiosis. Mol Cell, 31, 313–323. 18691964 10.1016/j.molcel.2008.05.021 PMC2584117

[24] Kaur, H. , De Muyt, A. and Lichten, M. (2015) Top3‐Rmi1 DNA single‐strand decatenase is integral to the formation and resolution of meiotic recombination intermediates. Mol Cell, 57, 583–594. 25699707 10.1016/j.molcel.2015.01.020 PMC4338413

[25] Zakharyevich, K. , Ma, Y. , Tang, S. , Hwang, P.Y. , Boiteux, S. and Hunter, N. (2010) Temporally and biochemically distinct activities of Exo1 during meiosis: double‐strand break resection and resolution of double Holliday junctions. Mol Cell, 40, 1001–1015. 21172664 10.1016/j.molcel.2010.11.032 PMC3061447

[26] Mancera, E. , Bourgon, R. , Brozzi, A. , Huber, W. and Steinmetz, L.M. (2008) High‐resolution mapping of meiotic crossovers and non‐crossovers in yeast. Nature, 454, 479–485. 18615017 10.1038/nature07135 PMC2780006

[27] Wei, W. , McCusker, J.H. , Hyman, R.W. , Jones, T. , Ning, Y. , Cao, Z. , Gu, Z. , Bruno, D. , Miranda, M. , Nguyen, M. *et al* (2007) Genome sequencing and comparative analysis of Saccharomyces cerevisiae strain YJM789. Proc Natl Acad Sci U S A, 104, 12825–12830. 17652520 10.1073/pnas.0701291104 PMC1933262

[28] Chen, S.Y. , Tsubouchi, T. , Rockmill, B. , Sandler, J.S. , Richards, D.R. , Vader, G. , Hochwagen, A. , Roeder, G.S. and Fung, J.C. (2008) Global analysis of the meiotic crossover landscape. Dev Cell, 15, 401–415. 18691940 10.1016/j.devcel.2008.07.006 PMC2628562

[29] Oke, A. , Anderson, C.M. , Yam, P. and Fung, J.C. (2014) Controlling meiotic recombinational repair ‐ specifying the roles of ZMMs, Sgs1 and Mus81/Mms4 in crossover formation. PLoS Genet, 10, e1004690. 25329811 10.1371/journal.pgen.1004690 PMC4199502

[30] Martini, E. , Borde, V. , Legendre, M. , Audic, S. , Regnault, B. , Soubigou, G. , Dujon, B. and Llorente, B. (2011) Genome‐wide analysis of heteroduplex DNA in mismatch repair‐deficient yeast cells reveals novel properties of meiotic recombination pathways. PLoS Genet, 7, e1002305. 21980306 10.1371/journal.pgen.1002305 PMC3183076

[31] Gresham, D. , Ruderfer, D.M. , Pratt, S.C. , Schacherer, J. , Dunham, M.J. , Botstein, D. and Kruglyak, L. (2006) Genome‐wide detection of polymorphisms at nucleotide resolution with a single DNA microarray. Science, 311, 1932–1936. 16527929 10.1126/science.1123726

[32] Qi, J. , Wijeratne, A.J. , Tomsho, L.P. , Hu, Y. , Schuster, S.C. and Ma, H. (2009) Characterization of meiotic crossovers and gene conversion by whole‐genome sequencing in Saccharomyces cerevisiae. BMC Genomics, 10, 475. 19832984 10.1186/1471-2164-10-475 PMC2770529

[33] Dutta, A. , Lin, G. , Pankajam, A.V. , Chakraborty, P. , Bhat, N. , Steinmetz, L.M. and Nishant, K.T. (2017) Genome Dynamics of Hybrid Saccharomyces cerevisiae During Vegetative and Meiotic Divisions. G3 (Bethesda), 7, 3669–3679. 28916648 10.1534/g3.117.1135 PMC5677154

[34] Schacherer, J. , Shapiro, J.A. , Ruderfer, D.M. and Kruglyak, L. (2009) Comprehensive polymorphism survey elucidates population structure of Saccharomyces cerevisiae. Nature, 458, 342–345. 19212320 10.1038/nature07670 PMC2782482

[35] Liti, G. , Carter, D.M. , Moses, A.M. , Warringer, J. , Parts, L. , James, S.A. , Davey, R.P. , Roberts, I.N. , Burt, A. , Koufopanou, V. *et al* (2009) Population genomics of domestic and wild yeasts. Nature, 458, 337–341. 19212322 10.1038/nature07743 PMC2659681

[36] Levy, S.E. and Myers, R.M. (2016) Advancements in Next‐Generation Sequencing. Annu Rev Genomics Hum Genet, 17, 95–115. 27362342 10.1146/annurev-genom-083115-022413

[37] Reis‐Filho, J.S. (2009) Next‐generation sequencing. Breast Cancer Res, 11 Suppl 3, S12. 20030863 10.1186/bcr2431 PMC2797692

[38] Anderson, C.M. , Chen, S.Y. , Dimon, M.T. , Oke, A. , DeRisi, J.L. and Fung, J.C. (2011) ReCombine: a suite of programs for detection and analysis of meiotic recombination in whole‐genome datasets. PLoS One, 6, e25509. 22046241 10.1371/journal.pone.0025509 PMC3201961

[39] Lao, J.P. and Hunter, N. (2010) Trying to avoid your sister. PLoS Biol, 8, e1000519. 20976046 10.1371/journal.pbio.1000519 PMC2957405

[40] Schwacha, A. and Kleckner, N. (1997) Interhomolog bias during meiotic recombination: meiotic functions promote a highly differentiated interhomolog‐only pathway. Cell, 90, 1123–1135. 9323140 10.1016/s0092-8674(00)80378-5

[41] Callender, T.L. , Laureau, R. , Wan, L. , Chen, X. , Sandhu, R. , Laljee, S. , Zhou, S. , Suhandynata, R.T. , Prugar, E. , Gaines, W.A. *et al* (2016) Mek1 Down Regulates Rad51 Activity during Yeast Meiosis by Phosphorylation of Hed1. PLoS Genet, 12, e1006226. 27483004 10.1371/journal.pgen.1006226 PMC4970670

[42] Joshi, N. , Brown, M.S. , Bishop, D.K. and Borner, G.V. (2015) Gradual implementation of the meiotic recombination program via checkpoint pathways controlled by global DSB levels. Mol Cell, 57, 797–811. 25661491 10.1016/j.molcel.2014.12.027 PMC4392720

[43] Lao, J.P. , Cloud, V. , Huang, C.C. , Grubb, J. , Thacker, D. , Lee, C.Y. , Dresser, M.E. , Hunter, N. and Bishop, D.K. (2013) Meiotic crossover control by concerted action of Rad51‐Dmc1 in homolog template bias and robust homeostatic regulation. PLoS Genet, 9, e1003978. 24367271 10.1371/journal.pgen.1003978 PMC3868528

[44] Falconer, E. , Hills, M. , Naumann, U. , Poon, S.S. , Chavez, E.A. , Sanders, A.D. , Zhao, Y. , Hirst, M. and Lansdorp, P.M. (2012) DNA template strand sequencing of single‐cells maps genomic rearrangements at high resolution. Nat Methods, 9, 1107–1112. 23042453 10.1038/nmeth.2206 PMC3580294

[45] Claussin, C. , Porubsky, D. , Spierings, D.C. , Halsema, N. , Rentas, S. , Guryev, V. , Lansdorp, P.M. and Chang, M. (2017) Genome‐wide mapping of sister chromatid exchange events in single yeast cells using Strand‐seq. Elife, 6. 10.7554/eLife.30560 PMC5734873 29231811

[46] Robinson, J.T. , Thorvaldsdottir, H. , Winckler, W. , Guttman, M. , Lander, E.S. , Getz, G. and Mesirov, J.P. (2011) Integrative genomics viewer. Nat Biotechnol, 29, 24–26. 21221095 10.1038/nbt.1754 PMC3346182

[47] Thorvaldsdottir, H. , Robinson, J.T. and Mesirov, J.P. (2013) Integrative Genomics Viewer (IGV): high‐performance genomics data visualization and exploration. Brief Bioinform, 14, 178–192. 22517427 10.1093/bib/bbs017 PMC3603213

[48] Karolchik, D. , Hinrichs, A.S. and Kent, W.J. (2012) The UCSC Genome Browser. Curr Protoc Bioinformatics, **Chapter 1**, Unit1 4. 10.1002/0471250953.bi0104s28 PMC2834533 19957273

[49] Lichten, M. (2008) Genomics: Thoroughly modern meiosis. Nature, 454, 421–422. 18650909 10.1038/454421a

[50] Mancera, E. , Bourgon, R. , Huber, W. and Steinmetz, L.M. (2011) Genome‐wide survey of post‐meiotic segregation during yeast recombination. Genome Biol, 12, R36. 21481229 10.1186/gb-2011-12-4-r36 PMC3218862

[51] Rockmill, B. , Lefrancois, P. , Voelkel‐Meiman, K. , Oke, A. , Roeder, G.S. and Fung, J.C. (2013) High throughput sequencing reveals alterations in the recombination signatures with diminishing Spo11 activity. PLoS Genet, 9, e1003932. 24204324 10.1371/journal.pgen.1003932 PMC3814317

[52] Anderson, C.M. , Oke, A. , Yam, P. , Zhuge, T. and Fung, J.C. (2015) Reduced Crossover Interference and Increased ZMM‐Independent Recombination in the Absence of Tel1/ATM. PLoS Genet, 11, e1005478. 26305689 10.1371/journal.pgen.1005478 PMC4549261

[53] Duroc, Y. , Kumar, R. , Ranjha, L. , Adam, C. , Guerois, R. , Md Muntaz, K. , Marsolier‐Kergoat, M.C. , Dingli, F. , Laureau, R. , Loew, D. *et al* (2017) Concerted action of the MutLbeta heterodimer and Mer3 helicase regulates the global extent of meiotic gene conversion. Elife, 6. 10.7554/eLife.21900 PMC5215242 28051769

[54] Krishnaprasad, G.N. , Anand, M.T. , Lin, G. , Tekkedil, M.M. , Steinmetz, L.M. and Nishant, K.T. (2015) Variation in crossover frequencies perturb crossover assurance without affecting meiotic chromosome segregation in Saccharomyces cerevisiae. Genetics, 199, 399–412. 25467183 10.1534/genetics.114.172320 PMC4317650

[55] Chakraborty, P. , Pankajam, A.V. , Lin, G. , Dutta, A. , Krishnaprasad, G.N. , Tekkedil, M.M. , Shinohara, A. , Steinmetz, L.M. and Nishant, K.T. (2017) Modulating Crossover Frequency and Interference for Obligate Crossovers in Saccharomyces cerevisiae Meiosis. G3 (Bethesda), 7, 1511–1524. 28315832 10.1534/g3.117.040071 PMC5427503

[56] Al‐Sweel, N. , Raghavan, V. , Dutta, A. , Ajith, V.P. , Di Vietro, L. , Khondakar, N. , Manhart, C.M. , Surtees, J.A. , Nishant, K.T. and Alani, E. (2017) mlh3 mutations in baker's yeast alter meiotic recombination outcomes by increasing noncrossover events genome‐wide. PLoS Genet, 13, e1006974. 28827832 10.1371/journal.pgen.1006974 PMC5578695

[57] Brion, C. , Legrand, S. , Peter, J. , Caradec, C. , Pflieger, D. , Hou, J. , Friedrich, A. , Llorente, B. and Schacherer, J. (2017) Variation of the meiotic recombination landscape and properties over a broad evolutionary distance in yeasts. PLoS Genet, 13, e1006917. 28763437 10.1371/journal.pgen.1006917 PMC5554000

[58] Laureau, R. , Loeillet, S. , Salinas, F. , Bergstrom, A. , Legoix‐Ne, P. , Liti, G. and Nicolas, A. (2016) Extensive Recombination of a Yeast Diploid Hybrid through Meiotic Reversion. PLoS Genet, 12, e1005781. 26828862 10.1371/journal.pgen.1005781 PMC4734685

[59] Wang, J. , Fan, H.C. , Behr, B. and Quake, S.R. (2012) Genome‐wide single‐cell analysis of recombination activity and de novo mutation rates in human sperm. Cell, 150, 402–412. 22817899 10.1016/j.cell.2012.06.030 PMC3525523

[60] Lu, S. , Zong, C. , Fan, W. , Yang, M. , Li, J. , Chapman, A.R. , Zhu, P. , Hu, X. , Xu, L. , Yan, L. *et al* (2012) Probing meiotic recombination and aneuploidy of single sperm cells by whole‐genome sequencing. Science, 338, 1627–1630. 23258895 10.1126/science.1229112 PMC3590491

[61] Wijnker, E. , Velikkakam James, G. , Ding, J. , Becker, F. , Klasen, J.R. , Rawat, V. , Rowan, B.A. , de Jong, D.F. , de Snoo, C.B. , Zapata, L. *et al* (2013) The genomic landscape of meiotic crossovers and gene conversions in Arabidopsis thaliana. Elife, 2, e01426. 10.7554/eLife.01426 PMC3865688 24347547

[62] Li, X. , Li, L. and Yan, J. (2015) Dissecting meiotic recombination based on tetrad analysis by single‐microspore sequencing in maize. Nat Commun, 6, 6648. 25800954 10.1038/ncomms7648 PMC4383000

